# Studying the role of cooperative hydration in stabilizing folded protein states

**DOI:** 10.1016/j.jsb.2016.09.003

**Published:** 2016-12

**Authors:** David J. Huggins

**Affiliations:** Theory of Condensed Matter Group, Cavendish Laboratory, University of Cambridge, 19 J J Thomson Avenue, Cambridge CB3 0HE, United Kingdom; Department of Chemistry, University of Cambridge, Lensfield Road, Cambridge, CB2 1EW, United Kingdom

**Keywords:** Protein folding, Molecular dynamics, Hydration

## Abstract

Understanding and modelling protein folding remains a key scientific and engineering challenge. Two key questions in protein folding are (1) why many proteins adopt a folded state and (2) how these proteins transition from the random coil ensemble to a folded state. In this paper we employ molecular dynamics simulations to address the first of these questions. Computational methods are well-placed to address this issue due to their ability to analyze systems at atomic-level resolution. Traditionally, the stability of folded proteins has been ascribed to the balance of two types of intermolecular interactions: hydrogen-bonding interactions and hydrophobic contacts. In this study, we explore a third type of intermolecular interaction: cooperative hydration of protein surface residues. To achieve this, we consider multiple independent simulations of the villin headpiece domain to quantify the contributions of different interactions to the energy of the native and fully extended states. In addition, we consider whether these findings are robust with respect to the protein forcefield, the water model, and the presence of salt. In all cases, we identify many cooperatively hydrated interactions that are transient but energetically favor the native state. Whilst further work on additional protein structures, forcefields, and water models is necessary, these results suggest a role for cooperative hydration in protein folding that should be explored further. Rational design of cooperative hydration on the protein surface could be a viable strategy for increasing protein stability.

## 1 Introduction

Two important questions have been identified in protein folding (Creighton, 1985). First, why do many proteins adopt folded conformations rather than forming an ensemble of unfolded conformations? Second, how do folded proteins transition from an ensemble of unfolded conformations to the correct folded conformation? In this paper we address the first question of why proteins fold. More accurately, we attempt to identify why currently-used computational models predict that proteins fold. The two questions above have been studied extensively using experimental and computational techniques (Anfinsen and Scheraga, 1975, Baker, 2000, Ben-Naim, 1991, Best and Hummer, 2010, Bryngelson et al., 1995, Dill, 1990, Dobson et al., 1998, Eisenberg and McLachlan, 1985, Fersht et al., 1992, Hartl and Hayer-Hartl, 2009, Kauzmann, 1959, Levinthal, 1968, Levy and Onuchic, 2006, Martin et al., 1991, Miranker et al., 1993, Nicholls et al., 1991) (Berger and Leighton, 1998, Duan and Kollman, 1998, Freddolino et al., 2010, Levitt, 1976, Levitt and Warshel, 1975, Li and Scheraga, 1987, Pande et al., 2003, Piana et al., 2011, Shaw et al., 2010, Sippl, 1993, Snow et al., 2002, Sugita and Okamoto, 1999, Voelz et al., 2010, Wang and Wang, 1999). Studies on protein-folding kinetics have highlighted the importance of intermediate states (Ptitsyn et al., 1990), cooperativity (Dill et al., 1993), free energy barriers (Shastry and Roder, 1998), and folding funnels (Bryngelson et al., 1995). Studies on protein-folding thermodynamics have focused mainly on the balance between two (non-mutually exclusive) types of intermolecular interactions (Ben-Naim, 1991, Dill, 1990, Hendsch and Tidor, 1994, Lazaridis et al., 1995, Myers and Pace, 1996, Pace et al., 1996, Pace et al., 2011, Pace et al., 2014, Strickler et al., 2006):

1) Hydrogen-bonding/polar interactions – Folded states commonly exhibit numerous hydrogen-bonding interactions between protein residues (McDonald and Thornton, 1994). However, these must compete with hydrogen-bonding interactions between the protein and the surrounding water and thus the balance of terms must be considered (Ben-Naim, 1991, Fernández et al., 2002, Fersht et al., 1985, Levy and Onuchic, 2006).
2) Hydrophobic/non-polar interactions – Folded states also exhibit numerous non-polar interactions between the protein residues (Nicholls et al., 1991). Again, these must compete with interactions between the protein and the surrounding water.

**Fig. 1:**
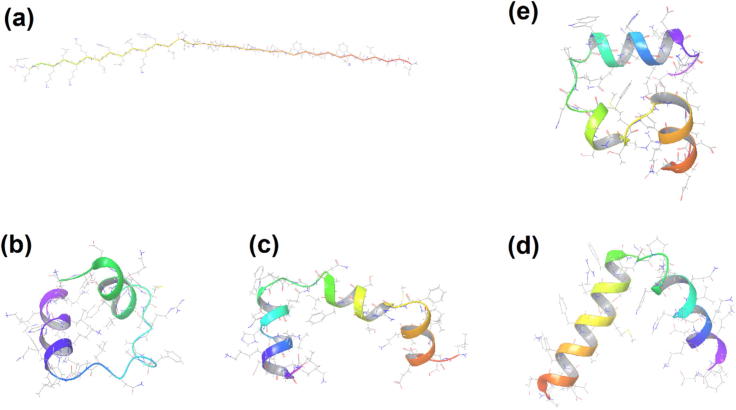
(a) Extended, (b–d) intermediate, and (e) native states of the villin headpiece domain.

In this work we consider a third mechanism in addition to those above: cooperative hydration of protein surface residues. The presence of water at the protein surface leads to a complex network of hydrogen bonds, with water molecules bridging interactions between protein residues. It has been suggested that this allows residues to be mutually solvated, forming strong cooperative interactions (Ben-Naim, 2011, Busch et al., 2013). Cooperative hydration effects have been identified as important in numerous other contexts (Guzmán et al., 2006, Okada and Tanaka, 2005, Pastorczak et al., 2013, Schellman, 1987). Thus, we are particularly interested in the prevalence of cooperative hydration and its effect on protein folding. Whilst analyses of polar and non-polar contributions to the energy and free energy of protein folding have been performed previously (Lazaridis et al., 1995, Makhatadze and Privalov, 1993, Privalov and Makhatadze, 1993, Robertson and Murphy, 1997), to our knowledge this is the first study that considers the difference between direct and water-mediated hydrogen-bonding interactions. We address this by considering the three mechanisms described above using average energies from long-timescale molecular dynamics (MD) simulations (Fenley et al., 2014, Roy et al., 2014). As a test case, we consider the villin headpiece, one of the mainstays of protein-folding studies (Duan et al., 1998, Fernández et al., 2003, Freddolino and Schulten, 2009, Jayachandran et al., 2006, Kubelka et al., 2003, Lee et al., 2000, McKnight et al., 1996, Mittal and Best, 2010, Wang et al., 2003). We explore the native state, a fully extended state, and three intermediate states (see Fig. 1).

To augment this analysis, we also consider the role of water using inhomogeneous fluid solvation theory (IFST), a statistical mechanical method that considers the contribution of individual hydration sites to the free-energy of the system. IFST has been used previously to study small-molecule hydration (Huggins, 2014b, Huggins and Payne, 2013, Lazaridis, 2000), protein-ligand binding (Haider and Huggins, 2013, Li and Lazaridis, 2005, Young et al., 2007), and artificial host-guest systems (Nguyen et al., 2012). We are also interested in how the choice of system treatment affects the predictions. Thus, we consider the effect of different water models, different force fields, and the presence or absence of salt.

## 2 Methods

### 2.1 System setup

We model five states of the of villin headpiece domain (McKnight et al., 1996): one native, one extended, and three intermediate states. The native state of the villin headpiece N68H mutant was taken from PDBID 1YRF (Chiu et al., 2005). An extended protein structure was generated from the protein sequence using Schrödinger’s peptide builder script, setting the phi angles to −71.6° and the psi angles to 135° for proline residues and the phi angles to −135° and the psi angles to 135° for all other residues (Zagrovic et al., 2002). The use of a fully extended state allows us to compare the native structure to a structure in which protein-water interactions are maximized. This is useful because it allows us to assess the balance of protein-protein and water-water contacts in the native state with protein-water interactions in the extended state. However, it is important to note that this will likely to lead to an overestimate of the total energy difference between the folded and unfolded states. The structures of the three intermediate states were taken from the study of Freddolino and Schulten (2009) which was generated using the CHARMM22 forcefield (Mackerell et al., 2004) with 200 mM sodium and chloride ions and TIP3P water (Jorgensen et al., 1983). In addition to multiple states, we considered a number of different treatments of the system. A list of the simulations performed is shown in Table 1.

Each starting structure was first subjected to gradient minimization with the appropriate CHARMM energy function using NAMD for 100,000 steps. We used the CMAP correction for CHARMM22. The systems were then neutralized with two chloride ions. The sodium and chloride ions were assigned parameters from Lamoureux and Roux (Lamoureux and Roux, 2006). The next stage in preparation was to generate a large water shell around each system with the SOLVATE program version 1.0 from the Max Planck Institute (Grubmüller and Groll, 1996). Water molecules were modelled with the TIP3P (Jorgensen et al., 1983) or TIP4P-2005 water model (Abascal and Vega, 2005). The resulting water globules were then cut to orthorhombic unit cells with side lengths of 42.0 Å × 42.0 Å × 147.0 Å for the extended state and 64.0 Å × 64.0Å × 64.0 Å for the other states. For each system treatment, the number of water molecules was adjusted to be equal for each of the states (Fenley et al., 2014). To standardize the geometries of the water molecules, every hydrogen atom was deleted and all the necessary hydrogen atoms and lone pairs were built using the appropriate geometry for the TIP3P and TIP4P-2005 models (oxygen-hydrogen bond lengths of 0.9572 Å, oxygen-lone pair bond lengths of 0.1546 Å, hydrogen-oxygen-lone pair bond angles of 52.26°, and hydrogen-oxygen-hydrogen bond angles of 104.52°). Where required, 32 sodium ions and 32 chloride ions were added to generate 200 mM salt solutions.

### 2.2 Molecular dynamics simulations

The next step was to generate independent protein conformations for each protein state and system treatment. After random initialization of velocities, equilibration was performed for 1.0 ns in an NPT ensemble at 300 K and 1 atm using Langevin temperature control and Nosé-Hoover (Martyna et al., 1994) Langevin piston pressure control (Feller et al., 1995). All systems were brought to equilibrium before continuing, by verifying that the energy fluctuations were stable. MD simulations were performed using an MD time step of 2.0 fs. Electrostatic interactions were modelled with a uniform dielectric and a dielectric constant of 1.0 throughout the equilibration and production runs. Van der Waals interactions were truncated at 11.0 Å with switching from 9.0 Å. Electrostatics were modelled using the particle mesh Ewald method (Essmann et al., 1995) and the systems were treated using orthorhombic periodic boundary conditions (PBC). Bonds to hydrogen were fixed using the SHAKE algorithm (Ryckaert et al., 1977) for the protein and the SETTLE algorithm (Miyamoto and Kollman, 1992) for waters. It is important to note that this approximation leaves water molecules as rigid bodies and precludes a rigorous assessment of vibrational entropy changes. 40.0 ns of production simulation were then performed at 300 K and 1 atm, saving system snapshots every 1.0 ns to yield forty snapshots in total for each protein state and system treatment. For each snapshot, atomic velocities were then reinitialized randomly to generate forty independent trajectories. Each protein state and system treatment was simulated twice. The first simulation was in the NPT ensemble with harmonic restraints on the two most distal alpha carbon atoms with a force constant of 1.0 kcal/mol/Å^2^ to bias the simulations to the states of interest. Analysis of the trajectories confirms that the appropriate structures are maintained for all system snapshots for all states. The second simulation was in the NVT ensemble with all atoms fixed. We then performed 1 ns of equilibration and 10 ns of production simulation for each protein state, system treatment, and ensemble. System setup, equilibration, and dynamics were performed using the Solvaware package on ARCHER. All MD simulations were performed using NAMD (Phillips et al., 2005) version 2.9.

### 2.3 System energies

Keeping the number of particles of each type constant, the difference in energy between a given state and the native state is simple to compute from the simulation (Fenley et al., 2014, Roy et al., 2014).

(1) $$\Delta E_{\mathrm{native}}=E_{\mathrm{state}}-E_{\mathrm{native}}$$

For all energy evaluations, we compute the energy of each state as an average of 100 snapshots in the NPT ensemble and report the average and standard deviation of the forty replicate simulations. The energy can be converted to an enthalpy by including the pressure multiplied by the volume change. As well as the total energy, we monitor the individual bonded and non-bonded components of the energy.

### 2.4 Atom energies

We considered the energy contribution from each individual atom (ΔE_atom_) by considering the total interaction energy in the native state and the extended state:

(2) $$\Delta E_{\mathrm{atom}}=E_{\mathrm{atom}}(\mathrm{native})-E_{\mathrm{atom}}(\mathrm{extended})$$

(3) $$E_{\mathrm{atom}}=1/2\overset{N}{\underset{j=1\text{,}j\neq a}{\sum }}E_{\mathrm{aj}}$$

The atom energy for a given state (E_atom_) is calculated as the sum of the non-bonded interaction energies between the atom (a) and all the other atoms in the system (N). We defined polar atoms as those with a partial charge greater than or equal to 0.3 and non-polar atoms as those with a partial charge less than 0.3. In the CHARMM forcefields, this divides all the traditional hydrogen bonding atoms (hydroxyl groups, carbonyl groups, amide groups, carboxylate groups, amine groups, and guanidine groups) from the traditional non hydrogen-bonding groups (non-polar carbon atoms, sulfur atoms, aliphatic hydrogen atoms, aromatic hydrogen atoms, and proline nitrogen atoms).

### 2.5 Hydrogen-bonding interaction energies

We considered the energy contribution from hydrogen-bonding interactions in the following manner. We defined hydrogen bond donor atoms as oxygen or nitrogen atoms connected to a hydrogen. We defined hydrogen bond acceptor atoms as oxygen or nitrogen atoms with an available lone pair. We defined a hydrogen bond in the following manner:

• Hydrogen bond donor and a hydrogen bond acceptor heavy atoms separated by a distance of 3.2 Å or less (Baker and Hubbard, 1984).
• An angle between the hydrogen bond donor atom, the hydrogen atom, and the hydrogen bond acceptor atom of 130° or more.

Both criteria must be met for a hydrogen bond to be present. For each system treatment, we identified the hydrogen bonds with an average persistence of greater than 50% across all 40 replicates of the native state simulation. Only these are considered. We then computed the difference between the hydrogen bond energies (ΔE_HB_) in the native state and the extended state:

(4) $$\Delta E_{\mathrm{HB}}=E_{\mathrm{HB}}(\mathrm{native})-E_{\mathrm{HB}}(\mathrm{extended})$$

(5) $$E_{\mathrm{HB}}=1/2\underset{i=a\text{,}b\text{,}c\text{,}d}{\sum }\overset{N}{\underset{j=1\text{,}j\neq i}{\sum }}E_{\mathrm{ij}}$$

**Fig. 2:**
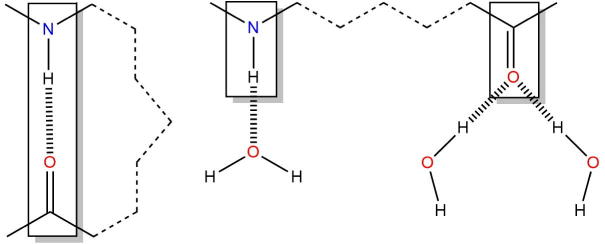
An illustration of the method to calculate the net contribution of a hydrogen bond to the energy of the native state relative to the extended state. Non-bonded interaction energies are calculated between the boxed atoms and all atoms in the system. The atoms form a hydrogen bond in the native state (left) and are typically exposed to solvent in the extended state (right).

The hydrogen bond energy for a given state (E_HB_) is calculated as the sum of the non-bonded interaction energies between (a) the donor atom, (b) the hydrogen atom, (c) the acceptor atom, (d) the atoms bonded to the acceptor atom and all the other atoms in the system (N). The basis for the calculation is depicted in Fig. 2.

The issue of molecular symmetry must also be addressed in these calculations. For example, the two oxygen atoms in a carboxylate residue (such as OD1 and OD2 in aspartate) can be exchanged by rotation of a torsion angle and are thus equivalent (indistinguishable). Thus, a hydrogen bond made by OD1 is equivalent to the same hydrogen bond made by OD2 and this constitutes only one hydrogen bond in total. This hydrogen bond is made if either of OD1 or OD2 pass the distance and angle cutoffs. For hydrogen bonds involving symmetry-related atoms, we calculate the distances between each symmetry related atom (such as OD1 and OD2) and their heavy atom partner in each frame. The shortest of these distances then define the atoms involved in the hydrogen bond for that frame.

### 2.6 Non-polar interaction energies

We considered the energy contribution from non-polar interactions in the following manner. We defined non-polar atoms as any carbon or sulfur atom not connected to an oxygen or nitrogen atom. We defined a non-polar interaction in the following manner:

• Two non-polar heavy atoms separated by a distance of 4.5 Å or less. This is the approximate distance at which the van der Waals interactions are 75% of their minimum value.

Contacts between atoms in the same residue and all 1–2, 1–3, and 1–4 atom-atom contacts are excluded to avoid spurious interactions. For each system treatment, we identified the non-polar interactions with an average persistence of greater than 50% across all 40 replicates of the native state simulation. Only these are considered further. We then computed the difference between the interaction energies (ΔE_NP_) in the native state and the extended state:

(6) $$\Delta E_{\mathrm{NP}}=E_{\mathrm{NP}}(\mathrm{native})-E_{\mathrm{NP}}(\mathrm{extended})$$

(7) $$E_{\mathrm{NP}}=1/2\underset{i=s\text{,}c\text{,}h}{\sum }\overset{N}{\underset{j=1\text{,}j\neq i}{\sum }}E_{\mathrm{ij}}$$

**Fig. 3:**
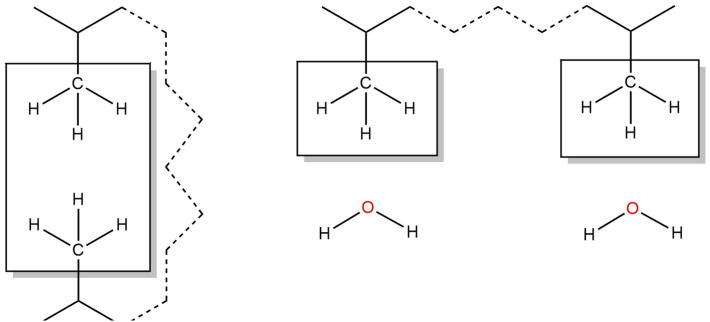
An illustration of the method to calculate the net contribution of a non-polar interaction to the energy of the native state relative to the extended state. Non-bonded interaction energies are calculated between the (boxed) carbon and hydrogen atoms and all atoms in the system. The atoms form a non-polar contact in the native state (left) and are exposed to solvent in the extended state (right).

The interaction energy for a given state (E_NP_) is calculated as the sum of the non-bonded interaction energies between the carbon (c) and hydrogen (h) or sulfur (s) atoms forming the interaction and all the atoms in the system (N). The calculation is depicted in Fig. 3.

Again, we respect molecular symmetry in these calculations, such that a non-polar interaction is present if any of the symmetry-related atoms pass the distance and angle cutoffs.

### 2.7 Cooperative hydration

We considered the energy contribution from cooperative hydration in the following manner. We defined hydrogen bond donor atoms as oxygen or nitrogen atoms connected to a hydrogen. We defined hydrogen bond acceptor atoms as oxygen or nitrogen atoms with an available lone pair. We defined a cooperatively hydrated interaction in the following manner:

• A hydrogen bond donor/hydrogen bond acceptor atom at a distance of 3.2–6.0 Å from another. hydrogen bond donor/ hydrogen bond acceptor atom.
• An oxygen atom from a single water molecule within 4.0 Å of each donor/acceptor atom.
• An angle between a hydrogen bond donor atom, the hydrogen atom, and the oxygen atom from the hydrating water molecule of 90° or more.
• An angle between a hydrogen bond acceptor atom, the heavy atom bonded to it, and the oxygen atom from the hydrating water molecule of 90° or more.

All criteria must be met for a hydrogen bond to be present. For each system treatment, we identified cooperatively hydrated interactions with an average persistence of greater than 50% across all 40 replicates of the native state simulation. Only these are considered further. We then computed the difference between the interaction energies (ΔE_CH_) in the native state and the extended state:

(8) $$\Delta E_{\mathrm{CH}}=E_{\mathrm{CH}}(\mathrm{native})-E_{CH}(\mathrm{extended})$$

(9) $$E_{\mathrm{CH}}=1/2\underset{i=a\text{,}b\text{,}c\text{,}d}{\sum }\overset{N}{\underset{j=1\text{,}j\neq i}{\sum }}E_{\mathrm{ij}}$$

**Fig. 4:**
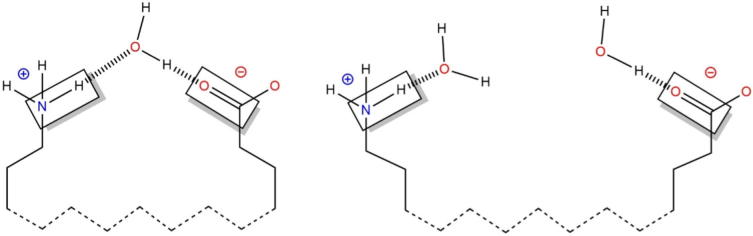
An illustration of the method to calculate the net contribution of cooperative hydration to the energy of the native state relative to the extended state. Non-bonded interaction energies are calculated between the boxed atoms and all atoms in the system. The atoms are cooperatively hydrated in the native state (left) and independently hydrated in the extended state (right).

The cooperative hydration energy for a given state (E_CH_) is calculated as the sum of the non-bonded interaction energies between the four atoms (a, b, c, d) and all the atoms in the system (N). The calculation is depicted in Fig. 4.

Again, we respect molecular symmetry in these calculations, such that cooperative hydration is present if any of the symmetry-related atoms pass the distance and angle cutoffs.

### 2.8 Hydration site calculations

We employ inhomogeneous fluid solvation theory (IFST) to calculate the contribution of individual hydration sites (ΔG_IFST_) to the system free energy (Huggins, 2015). Hydration sites were identified by clustering the water molecule positions from 5000 MD snapshots in the NVT ensemble in the context of a fixed protein structure. The free energies are calculated by combining the energetic (ΔE_IFST_) and entropic (ΔS_IFST_) contributions:

(10) $$\Delta G_{\mathrm{IFST}}=\Delta E_{\mathrm{IFST}}-T\Delta S_{\mathrm{IFST}}$$

ΔE_IFST_ is the difference between the average energy of a water molecule in the hydration site (E_site_) and a water molecule in the bulk (E_bulk_) (Huggins and Payne, 2013, Lazaridis, 1998a, Lazaridis, 1998b, Lazaridis, 2000, Nguyen et al., 2012).

(11) $$\Delta E_{\mathrm{IFST}}=E_{\mathrm{site}}-nE_{\mathrm{bulk}}$$

Calculated values of E_bulk_ are reported in Table 2. The free energy remains reasonably constant as the salt concentration increases, due to compensating changes in energy and enthalpy.

The number of snapshots and the sampling frequency are both important factors in reaching converged estimates (Huggins, 2012a, Huggins, 2012b, Huggins and Payne, 2013). To calculate ΔE_IFST_ we used 5000 snapshots and a sampling frequency of 2.0 ps. ΔS_IFST_ is calculated using the two-particle approximation, from the solute-water entropy (S_sw_), the water-water entropy (S_ww_), and the bulk water entropy (S_bulk_). The entropy change is computed in the reference frame of a fixed solute by separating the solvent and solute degrees of freedom:

(12) $$\Delta S_{\mathrm{IFST}}=S_{\mathrm{sw}}+S_{\mathrm{ww}}-{\mathrm{nS}}_{\mathrm{bulk}}$$

The S_sw_ terms were calculated using a k-nearest neighbors (KNN) approach (Huggins, 2014b). We use the first nearest neighbor (k = 1) in all cases (Singh et al., 2003). The nearest neighbor distance in translational space (d_trans_) between two water molecules is the Euclidean norm between the Cartesian coordinates of water molecule j in frame i and its nearest neighbor water molecule k in frame l. For correct treatment of waters near the periodic boundary, the minimum image convention is used (Adams, 1983, Smith, 1983, Smith, 1989). The nearest neighbor distance in orientational space (d_orient_) between two water molecules is the distance between the rotations required to bring the two orientations to the same reference orientation. The natural distance metric for the rotation group is twice the geodesic distance on the unit sphere (Huggins, 2014a). The quaternion representations of the rotations for water molecule j in frame i and its nearest neighbor water molecule k in frame l are denoted by q_ij_ and q_kl_. The total solute-water entropy can be estimated accurately by combining the translational and orientational distance metrics (Fogolari et al., 2015, Huggins, 2015).

(13) $$S_{\mathrm{sw}}=\mathrm{nR}\left\{\frac{1}{\mathrm{nF}}\overset{F}{\underset{i=1}{\sum }}\overset{n}{\underset{j=1}{\sum }}\ln\left[\frac{\pi d_{\mathrm{total}}^{6}\mathrm{nF}}{48V_{i}}\right]+\gamma \right\}$$

(14) $$d_{\mathrm{total}}=\sqrt{d_{\mathrm{trans}}^{2}+d_{\mathrm{orient}}^{2}}$$

(15) $$d_{\mathrm{trans}}=\sqrt{{\left(x_{\mathrm{ij}}-x_{\mathrm{kl}}\right)}^{2}+{\left(y_{\mathrm{ij}}-y_{\mathrm{kl}}\right)}^{2}+{\left(z_{\mathrm{ij}}-z_{\mathrm{kl}}\right)}^{2}}$$

(16) $$d_{\mathrm{orient}}=2\times \text{acos}(|{\text{q}}_{\text{ij}}.{\text{q}}_{\text{kl}}|)$$

R is the gas constant, F is the number of frames sampled, V_i_ is the volume of the system in frame i, and γ is Euler’s constant, which corrects for the asymptotic bias. S_ww_ was set to zero, as the majority of intermolecular correlation in the first hydration shell is expected to be captured by the S_sw_ term, as shown previously (Huggins, 2015). Calculated values of -TS_bulk_ are reported in Table 2. Whilst this approach does not capture protein motion, non-bonded protein interactions, or protein entropy changes, it is useful in highlighting surface water networks and cooperative binding effects.

## 3 Results

**Fig. 5:**
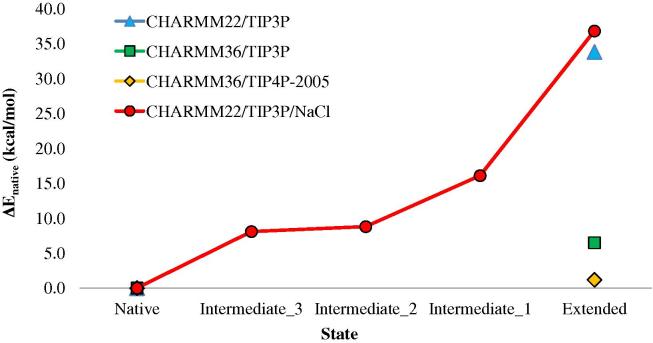
Differences in average energy between the native state and the alternative states for the four system treatments.

We began by calculating the relative energy of each state with respect to the native for each system treatment (see Fig. 5).

The native state is energetically favored in all four cases, but less favored in the case of the CHARMM36 force field. The intermediate states are, as expected, intermediate between the native and extended state energies. The relative energies are in excellent agreement (within 2.0 kcal/mol) for the simulations with and without NaCl. We also calculated the contribution of bonded and non-bonded energies to the total energies (Table 3).

As expected, the protein-protein and water-water interactions favor the native state, whilst the protein-water interactions favor the extended state. When summed, the interaction energies of the salt favor the extended state, presumably due to increased exposure of polar and charged atoms. The results are in broad agreement for all four treatments save that the contributions of the salt are understandably much smaller for the three cases where no additional ions were added (data not shown). We then moved on to explore the balance between polar and non-polar interactions, by considering which individual atoms contribute favorably or unfavorably to formation of the native state. Table 4 presents the result for the sum of all atoms, the sum of polar atoms, and the sum of non-polar atoms.

**Fig. 6:**
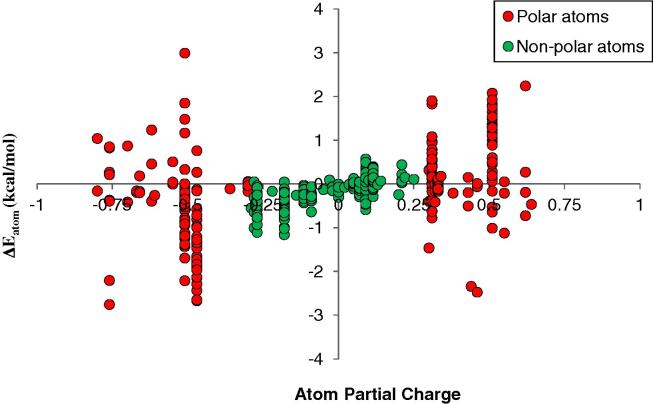
The distribution of atomic contributions to protein folding (in kcal/mol) versus the partial charge of the atom for the CHARMM22/TIP3P/NaCl treatment. Polar atoms are shown as red circles and non-polar atoms are shown as green circles.

This analysis yields the conclusion that polar and non-polar interactions make a net contribution to the protein folding energy that is very similar. This is true for all four system treatments. Fig. 6 presents the distribution of atomic contributions to the protein folding energy versus the partial charge of the atom.

There are more non-polar atoms (374) than polar atoms (208) and the magnitudes of the non-polar atom contributions are smaller than polar atoms. However, polar atoms make compensating favorable and unfavorable contributions. It is interesting to note that the negatively charged atoms tend to make a more favorable contribution to folding. However, this may be specific to villin, which has a net positive charge. We then used Eqs. (4), (5) to calculate the net contribution of hydrogen bonding interactions (as defined) to the native state with respect to the extended state. For the CHARMM22/TIP3P, CHARMM36/TIP3P, CHARMM36/TIP4P-2005, and CHARMM22/TIP3P/NaCl treatments, we identified 27, 30, 24, and 24 persistent hydrogen bonds in the native state. The contributions of these hydrogen bonds for the CHARMM22/TIP3P/NaCl treatment are reported in Table 5.

We find that the hydrogen bonding interactions tend to contribute favorably to formation of the native state, with a mean contribution of −0.8 kcal/mol (Table S1). However, there is a wide range of values, between favorable (−3.0 kcal/mol) and unfavorable (+2.1 kcal/mol). These findings are in reasonable agreement for all four system treatments (Table 6). However, hydrogen bonds contribute slightly less favorably to formation of the native state for the CHARMM36 forcefield and much less favorably again when the TIP4P-2005 water model is used.

We then used Eqs. (6), (7) to calculate the net contribution of non-polar interactions (as defined) to the native state with respect to the extended state. The contributions of these interactions for the CHARMM22/TIP3P/NaCl treatment are reported in Table 7.

We find that the non-polar interactions also contribute favorably to formation of the native state, with a mean contribution of −0.5 kcal/mol. These findings are in excellent agreement for all four system treatments, as shown in Table 8.

Taken together, these results suggest that a hydrogen-bonding interaction makes a greater contribution to the protein folding energy than a non-polar interaction. However, there are many more non-polar interactions. Thus, this analysis yields a similar conclusion to analysis of atom energies: that polar and non-polar interactions make a net contribution to the protein folding energy that is very similar. This is true for all four system treatments. We then considered the contributions of individual hydration sites surrounding the protein. First, we counted the number of hydration sites within 4.1 Å of the protein and the mean hydration site free energy calculated using IFST (see Table 9).

As expected, there are many more hydration sites around the extended state as the surface area is larger. However, we note that individual hydration sites around the extended state contribute less favorably to the free-energy than hydration sites around the native state. We also note that hydration sites for the CHARMM36 forcefield contribute more favorably to the free-energy than hydration sites for the CHARMM22 forcefield (using the TIP3P water model) and that TIP4P-2005 hydration sites contribute more favorably to the free-energy than TIP3P hydration sites (using the CHARMM36 forcefield). In particular, hydration sites around charged residues contribute significantly more favorably to the free-energy in the case of the TIP4P-2005 water model (data not shown). We then used Eqs. (8), (9) to calculate the net contribution of cooperative hydration (as defined) to the native state with respect to the extended state. For the CHARMM22/TIP3P, CHARMM36/TIP3P, CHARMM36/TIP4P-2005, and CHARMM22/TIP3P/NaCl treatments, we identified 17, 16, 59, and 15 persistent cooperatively hydrated interactions in the native state. The contributions of these cooperatively hydrated interactions for the CHARMM22/TIP3P/NaCl treatment are reported in Table 10.

We find that the cooperatively hydrated interactions tend to contribute favorably to formation of the native state, with a mean contribution of −0.2 kcal/mol. However, there is again a wide range of values, between favorable (−2.7 kcal/mol) and unfavorable (+0.7 kcal/mol). These findings are in reasonable agreement for all four system treatments (Table 11).

However, the significantly greater number of cooperatively hydrated interactions for the CHARMM36/TIP4P-2005 treatment means that cooperative hydration contributes much more favorably to formation of the native state in this case. It is important to note that direct hydrogen-bonding interactions tend to be more persistent than cooperatively hydrated interactions, but cooperatively hydrated interactions are far more common. For example, for the native conformation and the CHARMM22/TIP3P/NaCl treatment there are 127 direct hydrogen-bonding interactions but 438 cooperatively hydrated interactions. The number of hydrogen-bonding, non-polar, and cooperatively hydrated contacts in the native state are shown in Table 12.

**Fig. 7:**
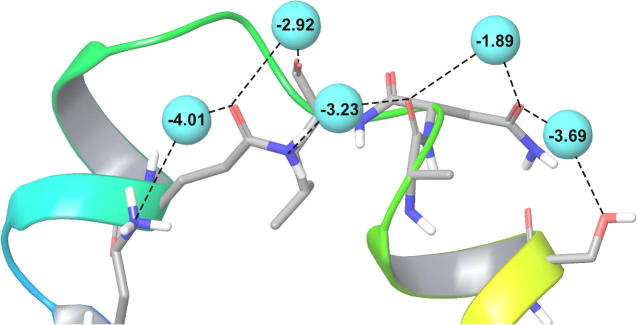
A network of cooperatively hydrated interactions around the native conformation of Villin for the CHARMM22/TIP3P/NaCl treatment. Hydration sites (cyan spheres) were identified by clustering a molecular dynamics simulation and are labelled by their contribution to the hydration free energy, calculated by IFST (in kcal/mol).

In each case, there are a large number of transient cooperatively-hydrated contacts. Analysis of hydration networks around a single protein conformations reveals that the surface is actually covered with cooperatively-hydrated interactions. These interactions can be explored by performing IFST calculations on the native state. An example of an extended network of cooperatively hydrated interactions is shown in Fig. 7.

**Fig. 8:**
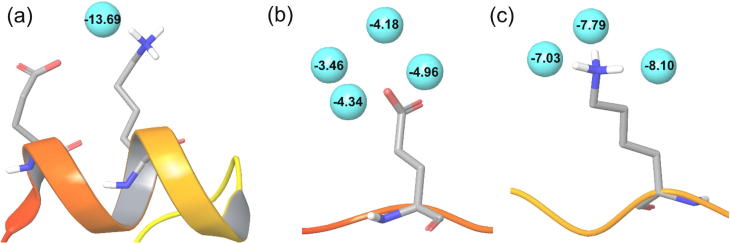
Hydration sites (cyan spheres) around Villin for the CHARMM22/TIP3P/NaCl treatment. (a) A cooperatively hydrated interaction between GLU45 and LYS48 in the native conformation, plus hydration sites around (b) GLU45 and (c) LYS48 in the extended conformation. Hydration sites were identified by clustering a molecular dynamics simulation and are labelled by their contribution to the hydration free energy, calculated by IFST (in kcal/mol).

It is also revealing to consider the free-energy contribution of a hydration site cooperatively hydrating a pair of residues around the native state and compare this with the free-energy contributions of hydration sites around the pair of residues in the extended state (Fig. 8).

The average contribution for hydration sites surrounding the independently-hydrated GLU45 and LYS48 are −4.24 kcal/mol and −7.64 kcal/mol. In combination they contribute −11.88 kcal/mol. The cooperatively-hydrated hydration site is extremely strongly bound and contributes -13.69 kcal/mol to the hydration free energy. Thus, the occurrence of cooperative hydration leads to more favorable hydration free energy and favors the native state.

## 4 Discussion

In this work we considered the importance of cooperative hydration (De Simone et al., 2005, Makhatadze and Privalov, 1994) in the protein folding energy of the villin headpiece. For comparison, we also considered the importance of intramolecular polar and non-polar protein interactions. To achieve this, we quantitatively assessed the balance between intramolecular protein interactions in the folded state and protein-water interactions in the extended state using mean energies from multiple MD simulations. To support the idea of cooperative hydration, we used IFST to study bridging hydration sites.

When comparing this study to previous work, it is important to discriminate the folding free energy from the folding enthalpy. Studies of folding enthalpy will be discussed first. Kubelka et al. determined the experimental folding enthalpy of the villin headpiece to be −27 kcal/mol (Kubelka et al., 2003). Pace et al. determined the experimental folding enthalpy to be -13 kcal/mol. We have derived this second estimate from the reported folding enthalpy of −31 kcal/mol at the melting temperature of 74.4 °C and the reported heat capacity of 0.374 kcal/mol/K (Pace et al., 2014). The folding energies of villin calculated here are in reasonable agreement (Fig. 5) with these values. The folding enthalpies for the CHARMM22/TIP3P, CHARMM36/TIP3P, CHARMM36/TIP4P-2005, and CHARMM22/TIP3P/NaCl treatments are −31 kcal/mol, −9 kcal/mol, −3 kcal/mol, and −38 kcal/mol respectively. However, as noted above, a direct comparison between calculation and experiment is not strictly appropriate as the average energy of the unfolded state ensemble is likely to differ from the energy of the fully extended state considered here. Based on our calculations, polar and non-polar interactions both contribute to the energetic stability of the folded state of villin. The findings are in agreement with work from Makhatadze and Privalov (Makhatadze et al., 1994) on the enthalpy of folding at 25 °C. It should be said that these authors, and others, find that protein folding can have a pronounced temperature dependence (Privalov and Makhatadze, 1990) and our simulations are performed at 25 °C only. From our work, approximately half of the villin folding energy is derived from polar interactions and half from non-polar interactions (Table 4). For the villin test case studied, individual hydrogen bonding interactions are predicted to make a similar contribution to individual non-polar contacts to the folding energy, but are less numerous. Makhatadze and Privalov have noted that different proteins may have a different balance of polar and non-polar effects (Makhatadze and Privalov, 1993).

The majority of protein folding studies have focused on protein stability, which is related to the free energy rather than the energy considered here. Experimental studies on 22 proteins suggest that hydrophobic interactions contribute 60% and hydrogen bonds contribute 40% to protein stability (Pace et al., 2011). Other experimental and theoretical studies are in reasonable agreement. (Myers and Pace, 1996, Pace et al., 1996, Pace et al., 2014) However, it is worth noting that stabilizing and destabilizing forces contributing to protein stability may be size-dependent (Pace et al., 2011) In contrast to this, some theoretical studies have suggested that hydrogen bonds contribute little, nothing, or actually unfavorably to protein stability (Campos et al., 2005, Lazaridis et al., 1995, Sippl et al., 1996). From experimental studies, hydrogen bonds have been estimated to contribute −1.0 kcal/mol (Fersht et al., 1985), −1.1 kcal/mol (Pace et al., 2014), and −0.5 kcal/mol (Bowie, 2011) to the folding free energy whereas non-polar contacts have been estimated to contribute −1.1 kcal/mol (Kellis et al., 1988) and −1.1 kcal/mol (Pace et al., 2011). For this work we calculate the contribution to the folding energy of a hydrogen bond to be −0.8 kcal/mol and a non-polar contact to be −0.5 kcal/mol. Pace and co-workers have studied specific mutations to villin and the effect on folding (Pace et al., 2011, Pace et al., 2014). For changes to polar residues, they identified SER43 and THR54 as being important for stability. Our calculations predict that THR54 makes a strong and persistent hydrogen bond and that this contributes favorably to the folding energy (Table 5). For changes to non-polar residues, they identified MET52, PHE57, and LEU60 as being important for stability. Our calculations predict that all three residues make strong and persistent non-polar interactions and contribute favorably to the folding energy (Table 7). We also identify only two persistent salt bridges, and both are predicted to energetically favor the folded state. Previous work suggests that salt bridges can stabilize or destabilize proteins (Hendsch and Tidor, 1994, Strickler et al., 2006). For hydrogen bonds, non-polar contacts, and cooperative hydration, we note that importance of considering the protein structural ensemble, as there are many transient interactions that contribute to the preference for the folded state.

The novelty of this work is the differentiation between direct and water-bridged hydrogen-bonding interactions. We identify a small number of strong and persistent intramolecular hydrogen bonds and a large number of weaker and more transient cooperatively hydrated interactions. The results suggests that cooperative hydration contributes favorably to the folding energy of the villin headpiece, supporting a hypothesis put forward by Ben Naim that mutual solvation is a driving force for protein folding (Ben-Naim, 2011). However, it is important to note that this analysis considers only energy differences and does not take into account entropy changes. It is only in combination that the energy and entropy deliver the most relevant quantity of the folding free energy. Further theoretical developments are needed in order to rigorously extract entropy changes from all-atom molecular simulations. Computing entropy changes will also necessitate effective consideration of an ensemble of protein structures. A number of groups have generated reference models of protein unfolded states of proteins in order to model protein folding. These have been used to study charge-charge interactions, (Zhou, 2002) backbone solvent accessibility, (Creamer et al., 1997), early folding events, (Felitsky et al., 2008) electrostatics (Kundrotas and Karshikoff, 2002), the pH dependence of protein stability (Elcock, 1999). In this work we have used a highly idealized unfolded state, which is fully extended. Whilst this allows us to consider the balance of protein-water interactions and intramolecular protein interactions, it does represent a highly simplified model of the unfolded state and thus the magnitude of the differences from the native state are unlikely to be accurate. However, the majority of protein residues are likely to be solvent exposed for the majority of conformations in the ensemble and thus the trends are likely to be meaningful. Moving along the continuum from the fully extended to the native state, the increased number of protein-protein non-polar, hydrogen bonding, and cooperatively hydrated interactions are all likely to contribute favorably to the energy.

For the major conclusions of the paper, we find similar result using two forcefields, two water models, and the presence or absence of 200 mM sodium chloride. However, it is important to note that this work studies why classical models of proteins fold in silico. Once this question has been addressed, we must then consider whether these classical models provide an accurate representation of hydrated proteins. As noted above, experimental data on alanine scanning agrees with the predictions for polar and non-polar contacts (Pace et al., 2011, Pace et al., 2014), but the equivalent data for cooperatively hydrated interactions is lacking. Mutation of villin residues such as GLU45, LYS48, LYS70, and LYS73, which are involved in bridged but not direct hydrogen bonding (see Table 10), could be used to assess the relative importance of cooperative hydration. We should also not that most of the work described here deals with energy differences only. Entropy is clearly important in protein folding and the solvent is of vital importance (Schäfer et al., 2001). It has been suggested that water at hydrophobic surfaces forms an entropically unfavorable hydration shell and thus that hydrophobic contacts have an inherent driving force. This is commonly referred to as the hydrophobic effect (Chandler, 2005, Muller, 1990, Sharp et al., 1991, Snyder et al., 2011, Spolar et al., 1989, Tsai et al., 1997, Zhou et al., 2004). However, entropy cannot typically be modelled as pairwise additive and cannot be assigned to individual atoms or interactions. Whilst IFST naturally includes solvation entropy, it does not consider the coupling between solvent and solute degrees of freedom. It is important to stress that attempting to derive understanding from considering the entropy of a subset of the system degrees of freedom is uninformative or actually misleading. Unfortunately, accurate calculations of entropy considering all the system degrees of freedom from all-atom simulations are many years away. Importantly, both hydrogen-bonding and non-polar interactions are cooperative, such that entropy is not additive (Zhou and Gilson, 2009). Rather, the formation of one interaction “favors” the formation of another (and vice versa). It would be interesting to explore the entropy of conformational ensembles in the context of protein folding using molecular simulation in future work (McClendon et al., 2012).

As well as providing an understanding of why proteins fold in classical molecular dynamics simulations, this work has implications for protein design. Firstly, it adds to the body of literature suggesting that mutations designed to form additional hydrogen bonds, salt bridges, and non-polar contacts are all reasonable strategies to stabilize the folded state. However, forming additional hydrogen bonds, salt bridges, or non-polar contacts may be difficult to engineer in the protein interior without disrupting the secondary structure. Conversely, mutations designed to take advantage of existing water networks at the protein surface may lead to increased stability due to the formation of cooperative hydration effects. This strategy also has the advantage that mutations naturally introduce polar residues, avoiding an increase in the number of non-polar residues, which can lead to aggregation. In practice, plots such as Fig. 6 could be used to identify residues that do not contribute favorably to the folding energy. Hydration site analysis using methods such as IFST could then be used to engineer bridged hydrogen bonding interactions by mutation of these residues. These predictions could be quantitatively assessed using methods such as free-energy perturbation. Further work is necessary to confirm whether this should be considered as a general design principle, as this study is limited to one test case and considers energy changes rather than the more desirable free-energy changes.

In summary, the results of this work highlight a potential role for cooperative hydration as a driving force in protein folding and suggest a number of villin mutations that can be used to test the prediction. Whilst this is not a definitive article on cooperative hydration or protein folding, it suggests that we should explicitly consider the importance of solvation effects in protein folding. We suggest that support from additional forcefields, water models, and protein test cases is needed to establish this as a general design principle. However, we propose that cooperative hydration could be useful in the context of rational protein design, because it leads naturally to mutations on the proteins surface and does not introduce hydrophobic residues which can contribute to aggregation.

## Conflict of Interest

The authors declare that they have no conflicts of interest with the contents of this article.

## Author Contributions

DJH conceived the idea for the project, performed the calculations, analyzed the results, and wrote the paper.

## Figures and Tables

**Table 1:** Protein states, system treatments, and simulations performed.

Forcefield	Water Model	NaCl (mM)	Extended	Intermediate 1	Intermediate 2	Intermediate 3	Native
CHARMM22	TIP3P	200	40 × 10 ns	40 × 10 ns	40 × 10 ns	40 × 10 ns	40 × 10 ns
CHARMM22	TIP3P	–	40 × 10 ns	–	–	–	40 × 10 ns
CHARMM36	TIP3P	–	40 × 10 ns	–	–	–	40 × 10 ns
CHARMM36	TIP4P-2005	–	40 × 10 ns	–	–	–	40 × 10 ns

**Table 2:** Thermodynamic properties of bulk water determined from (a) 5000 snapshots from a 10 ns simulation in the NPT ensemble and (b) bidirectional FEP calculations using 32 lambda windows with 25 ps of equilibration and 175 ps of production per window. Free energies (and the derived entropies) were calculated using a BAR estimator and the statistical error was less than 0.1 kcal/mol in each case.

Water model	NaCl (mM)	^a^E_bulk_	^b^G_bulk_	^a,b^-TS_bulk_
TIP3P	0	−9.8	−6.3	3.5
TIP3P	50	−10.2	−6.5	3.8
TIP3P	100	−10.6	−6.5	4.1
TIP3P	150	−11.1	−6.3	4.7
TIP3P	200	−11.4	−6.5	5.0
TIP4P-2005	0	−11.6	−7.0	4.6

**Table 3:** The contributions to components of the bonded and non-bonded energies for the native and extended states using the CHARMM22/TIP3P/NaCl system treatment.

Interaction	Component	E_Native_ (kcal/mol)	E_Extended_ (kcal/mol)	ΔE_native_ (kcal/mol)	Favoured state
Protein-protein	Bonded	573.8 ± 1.0	578.0 ± 1.2	−4.2	Native
Protein-protein	Non-bonded	−642.6 ± 29.6	−165.5 ± 24.5	−477.1	Native
Protein-water	Non-bonded	−1,465.2 ± 46.6	−2,106.7 ± 57.6	641.5	Extended
Water-water	Non-bonded	−77,156.9 ± 33.5	−76,920.8 ± 52.5	−236.1	Native
Protein-salt	Non-bonded	−179.1 ± 28.3	−367.2 ± 77.8	188.1	Extended
Water-salt	Non-bonded	−9,085.3 ± 46.5	−8,974.0 ± 61.2	−111.3	Native
Salt-salt	Non-bonded	−1,030.3 ± 20.8	−993.4 ± 28.8	−36.9	Native
Total		−88,985.7 ± 16.0	−88,949.6 ± 16.5	−36.1	Native

**Table 4:** The contribution of three sets of atoms to the energy difference between the native and extended states for each system treatment: all protein atoms, polar protein atoms, and non-polar protein atoms.

Treatment	ΔE_total_ (kcal/mol)	ΔE_polar_ (kcal/mol)	ΔE_non-polar_ (kcal/mol)
CHARMM22/TIP3P	−63.8	−34.3	−29.5
CHARMM36/TIP3P	−62.9	−31.3	−31.6
CHARMM36/TIP4P-2005	−49.7	−21.0	−28.7
CHARMM22/TIP3P/NaCl	−62.0	−31.5	−30.5

**Table 5:** The mean and standard deviation of the interaction energy for individual hydrogen bonds in the native and extended states for the CHARMM22/TIP3P/NaCl treatment, plus the energy difference. In each case, the donor and acceptor heavy atoms are reported, using residue names, residue numbers, and CHARMM atom names. Only the fifteen contacts with the greatest persistence are shown. The results for all persistent contacts can be found in Supplementary information Table S1.

Donor atom	Acceptor atom	Persistence	Native E_HB_ (kcal/mol)	Extended E_HB_ (kcal/mol)	ΔE_HB_ (kcal/mol)
PHE47 N	SER43 O	0.96 ± 0.06	−6.1 ± 0.3	−5.4 ± 0.2	−0.7
LYS48 N	ASP44 O	0.99 ± 0.01	3.9 ± 0.2	4.5 ± 0.3	−0.6
PHE51 N	PHE47 O	0.99 ± 0.01	0.3 ± 0.3	2.1 ± 0.2	−1.7
ARG55 NE	ASP44 OD1/ASP44 OD2	1.00 ± 0.00	−100.1 ± 0.2	−99.2 ± 0.4	−1.0
ARG55 NH1/ARG55 NH2	ASP44 OD1/ASP44 OD2	1.00 ± 0.00	−97.1 ± 0.0	−94.1 ± 0.4	−3.0
PHE58 N	THR54 O	1.00 ± 0.01	−8.7 ± 0.2	−7.8 ± 0.2	−1.0
ALA59 N	ARG55 O	0.96 ± 0.02	−2.2 ± 0.2	−0.8 ± 0.2	−1.4
GLN66 N	PRO62 O	0.99 ± 0.01	−4.9 ± 0.2	−3.9 ± 0.2	−0.9
GLN67 N	LEU63 O	1.00 ± 0.00	0.6 ± 0.2	1.1 ± 0.3	−0.5
HIS68 N	TRP64 O	0.99 ± 0.01	2.6 ± 0.2	3.8 ± 0.2	−1.2
LEU69 N	LYS65 O	0.99 ± 0.01	0.4 ± 0.2	1.5 ± 0.2	−1.1
LYS70 N	GLN66 O	0.99 ± 0.01	0.7 ± 0.2	1.7 ± 0.2	−1.0
LYS71 N	GLN67 O	0.99 ± 0.01	0.4 ± 0.3	1.8 ± 0.2	−1.4
GLU72 N	HIS68 O	0.99 ± 0.01	−2.0 ± 0.3	−0.9 ± 0.2	−1.1
LYS73 N	LEU69 O	0.95 ± 0.03	1.6 ± 0.2	1.3 ± 0.4	0.3
		Mean	−14.0	−13.0	−1.1

**Table 6:** The mean interaction energy for hydrogen bonds in the native and extended states for each system treatment, plus the resulting energy difference.

Treatment	Native E_HB_ (kcal/mol)	Extended E_HB_ (kcal/mol)	ΔE_HB_ (kcal/mol)
CHARMM22/TIP3P	−9.7	−8.9	−0.8
CHARMM36/TIP3P	−12.1	−11.4	−0.7
CHARMM36/TIP4P-2005	−11.2	−10.9	−0.3
CHARMM22/TIP3P/NaCl	−10.2	−9.4	−0.8

**Table 7:** The mean and standard deviation of the interaction energy for non-polar contacts in the native and extended states for the CHARMM22/TIP3P/NaCl treatment, plus the energy difference. In each case, the two non-polar heavy atoms are reported, using residue names, residue numbers, and CHARMM atom names. Only the fifteen contacts with the greatest persistence are shown. The results for all persistent contacts can be found in Supplementary information Table S2.

Atom 1	Atom 2	Persistence	Native E_NP_ (kcal/mol)	Extended E_NP_ (kcal/mol)	ΔE_NP_ (kcal/mol)
MET53 CE	LEU61 CD1/LEU61 CD2	1.10 ± 0.14	−11.4 ± 0.1	−10.7 ± 0.1	−0.7
LEU61 CD1/LEU61 CD2	LYS65 CB	1.11 ± 0.49	−8.4 ± 0.1	−7.5 ± 0.1	−0.9
PHE51 CE1/PHE51 CE2	LEU75 CD1/LEU75 CD2	1.13 ± 0.10	−6.1 ± 0.1	−5.9 ± 0.1	−0.3
MET53 SD	LEU61 CD1/LEU61 CD2	1.14 ± 0.12	−12.6 ± 0.1	−11.2 ± 0.1	−1.4
PHE47 CE1/PHE47 CE2	PHE58 CD1/PHE58 CD2	1.22 ± 0.12	0.0 ± 0.1	0.4 ± 0.1	−0.4
PHE51 CE1/PHE51 CE2	PHE58 CE1/PHE58 CE2	1.25 ± 0.22	3.1 ± 0.1	3.1 ± 0.1	0.0
VAL50 CG1/VAL50 CG2	PHE51 CE1/PHE51 CE2	1.28 ± 0.18	−2.0 ± 0.1	−1.7 ± 0.1	−0.3
VAL50 CG1/VAL50 CG2	PHE51 CD1/PHE51 CD2	1.31 ± 0.16	−4.7 ± 0.1	−4.4 ± 0.1	−0.4
PHE51 CD1/PHE51 CD2	PHE58 CE1/PHE58 CE2	1.34 ± 0.20	0.4 ± 0.1	0.4 ± 0.1	0.0
PHE58 CE1/PHE58 CE2	GLN66 CG	1.48 ± 0.12	−10.5 ± 0.1	−9.9 ± 0.1	−0.6
PHE47 CE1/PHE47 CE2	PHE51 CE1/PHE51 CE2	1.51 ± 0.08	2.9 ± 0.1	3.1 ± 0.1	−0.2
PHE47 CE1/PHE47 CE2	ARG55 CG	1.57 ± 0.12	−3.4 ± 0.1	−2.9 ± 0.1	−0.5
PHE47 CD1/PHE47 CD2	PHE51 CD1/PHE51 CD2	1.60 ± 0.13	−2.8 ± 0.1	−2.3 ± 0.1	−0.5
PHE47 CE1/PHE47 CE2	PHE51 CD1/PHE51 CD2	1.76 ± 0.22	0.1 ± 0.1	0.4 ± 0.1	−0.3
PHE47 CD1/PHE47 CD2	ARG55 CG	1.86 ± 0.15	−6.1 ± 0.1	−5.6 ± 0.1	−0.5
			−3.6	−3.1	−0.5

**Table 8:** The mean interaction energy for non-polar interactions in the native and extended states for each system treatment, plus the resulting energy difference.

Treatment	Native E_NP_ (kcal/mol)	Extended E_NP_ (kcal/mol)	ΔE_NP_ (kcal/mol)
CHARMM22/TIP3P	–3.6	−3.1	−0.5
CHARMM36/TIP3P	−3.8	−3.3	−0.5
CHARMM36/TIP4P-2005	−3.2	−2.7	−0.5
CHARMM22/TIP3P/NaCl	−4.0	−3.5	−0.5

**Table 9:** The average number of hydration sites within 4.1 Å of the protein surface for each –protein state and system treatment, along with the mean and standard deviation of the hydration site free energy.

Treatment	State	Hydration sites	ΔG_IFST_ (kcal/mol)	σ (kcal/mol)
CHARMM22/TIP3P	Native	442	−1.25	1.21
CHARMM22/TIP3P	Extended	875	−1.04	0.91
CHARMM36/TIP3P	Native	440	−1.84	1.62
CHARMM36/TIP3P	Extended	886	−1.42	1.25
CHARMM36/TIP4P-2005	Native	437	−2.21	1.66
CHARMM36/TIP4P-2005	Extended	871	−1.78	1.32
CHARMM22/TIP3P/NaCl	Native	360	−1.85	1.69
CHARMM22/TIP3P/NaCl	Intermediate 1	378	−1.79	1.56
CHARMM22/TIP3P/NaCl	Intermediate 2	433	−1.63	1.45
CHARMM22/TIP3P/NaCl	Intermediate 3	468	−1.58	1.48
CHARMM22/TIP3P/NaCl	Extended	708	−1.41	1.32

**Table 10:** The mean and standard deviation of the interaction energy for individual cooperatively hydrated interactions in the native and extended states for the CHARMM22/TIP3P/NaCl treatment, plus the energy difference. In each case, the donor or acceptor atoms are reported, using residue names, residue numbers, and CHARMM atom names.

Atom 1	Atom 2	Persistence	Native E_CH_ (kcal/mol)	Extended E_CH_ (kcal/mol)	ΔE_CH_ (kcal/mol)
LEU42 O	ARG55 HH11/HH21	0.63 ± 0.25	−67.22 ± 0.38	−68.4 ± 0.4	1.2
ASP44 HN	ASP44 OD1/OD2	0.58 ± 0.14	−34.01 ± 0.31	−31.3 ± 0.3	−2.7
LYS48 O	ALA49 O	0.64 ± 0.08	3.88 ± 0.30	3.9 ± 0.2	−0.1
ALA49 O	VAL50 O	0.92 ± 0.08	0.95 ± 0.22	0.8 ± 0.2	0.1
VAL50 O	LYS73 HZ1/HZ2/HZ3	0.85 ± 0.11	−5.18 ± 0.24	−5.3 ± 0.2	0.1
ALA59 O	LEU61 O	0.89 ± 0.05	17.08 ± 0.25	16.4 ± 0.2	0.7
ALA59 O	GLN66 HE21	0.87 ± 0.08	−16.43 ± 0.30	−16.9 ± 0.2	0.5
GLN66 OE1	LYS70 HZ1/HZ2/HZ3	0.68 ± 0.13	−31.91 ± 0.20	−31.2 ± 0.2	−0.7
LYS71 O	GLU72 O	0.62 ± 0.06	1.68 ± 0.20	0.9 ± 0.2	0.7
GLY74 O	PHE76 OT1/OT2	0.69 ± 0.14	−28.51 ± 0.38	−27.8 ± 0.3	−0.7
LEU75 O	PHE76 OT1/OT2	0.85 ± 0.10	−20.31 ± 0.33	−20.5 ± 0.3	0.2
ASP44 OD1/OD2	ARG55 HN	0.51 ± 0.20	−29.87 ± 0.26	−29.0 ± 0.4	−0.9
ASP44 OD1/OD2	SER56 HN	0.86 ± 0.16	−22.47 ± 0.23	−22.2 ± 0.4	−0.3
GLU72 OE1/OE2	LYS73 HZ1/HZ2/HZ3	0.61 ± 0.20	−23.59 ± 0.30	−23.2 ± 0.3	−0.4
GLU45 OE1/OE2	LYS48 HZ1/HZ2/HZ3	0.69 ± 0.31	−23.93 ± 0.72	−23.2 ± 0.3	−0.7
		Mean	−18.7	−18.5	−0.2

**Table 11:** The mean interaction energy for cooperatively hydrated interactions in the native and extended states for each system treatment, plus the resulting energy difference.

Treatment	Native E_CH_ (kcal/mol)	Extended E_CH_ (kcal/mol)	ΔE_CH_ (kcal/mol)
CHARMM22/TIP3P	−21.9	−21.5	−0.3
CHARMM36/TIP3P	−18.5	−18.1	−0.4
CHARMM36/TIP4P-2005	−21.1	−20.8	−0.3
CHARMM22/TIP3P/NaCl	−15.2	−14.9	−0.2

**Table 12:** The total number of hydrogen-bonding, non-polar, and cooperatively hydrated contacts in the native states for each treatment, along with the number of persistent contacts.

Treatment	Hydrogen bonding		Non-polar		Cooperatively hydrated	
	Total contacts	Persistent contacts	Total contacts	Persistent contacts	Total contacts	Persistent contacts
CHARMM22/TIP3P	142	27	607	72	474	17
CHARMM36/TIP3P	130	30	526	71	470	16
CHARMM36/TIP4P-2005	146	24	532	84	531	59
CHARMM27/TIP3P/NaCl	127	24	533	73	438	15
