# Supplementary material for: Studying the role of cooperative hydration in stabilizing folded protein states
Source: J Struct Biol. 2016 Dec;196(3):394–406. doi: 10.1016/j.jsb.2016.09.003 (PMC5131609; doi:10.1016/j.jsb.2016.09.003)
Supplement: Supplementary data [file mmc1.docx]

**Studying the Role of Cooperative Hydration in Stabilizing Folded Protein States**

**Supporting Information**

| **Donor Atom** | **Acceptor Atom** | **Persistence** | **Native**  **E_HB_ (kcal/mol)** | **Extended E_HB_ (kcal/mol)** | **ΔE_HB_ (kcal/mol)** |
| --- | --- | --- | --- | --- | --- |
| PHE47 N | SER43 O | 0.96 ± 0.06 | -6.1 ± 0.3 | -5.4±0.2 | -0.7 |
| LYS48 N | ASP44 O | 0.99 ± 0.01 | 3.9 ± 0.2 | 4.5±0.3 | -0.6 |
| ALA49 N | GLU45 O | 0.90 ± 0.04 | -2.6 ± 0.2 | -1.7±0.2 | -0.9 |
| VAL50 N | ASP46 O | 0.95 ± 0.03 | 7.8 ± 0.2 | 7.9±0.2 | -0.2 |
| PHE51 N | PHE47 O | 0.99 ± 0.01 | 0.3 ± 0.3 | 2.1±0.2 | -1.7 |
| GLY52 N | LYS48 O | 0.56 ± 0.07 | 7.6 ± 0.2 | 8.7±0.2 | -1.1 |
| ARG55 N | ASP44 OD1/ASP44 OD2 | 0.76 ± 0.21 | -29.9 ± 0.3 | -29.0±0.4 | -0.9 |
| ARG55 NE | ASP44 OD1/ASP44 OD2 | 1.00 ± 0.00 | -100.1 ± 0.2 | -99.2±0.4 | -1.0 |
| ARG55 NH1/ARG55 NH2 | ASP44 OD1/ASP44 OD2 | 1.00 ± 0.00 | -97.1 ± 0.0 | -94.1±0.4 | -3.0 |
| ALA57 N | THR54 OG1 | 0.93 ± 0.03 | -20.2 ± 0.4 | -19.2±0.3 | -1.0 |
| PHE58 N | THR54 O | 1.00 ± 0.01 | -8.7 ± 0.2 | -7.8±0.2 | -1.0 |
| ALA59 N | ARG55 O | 0.96 ± 0.02 | -2.2 ± 0.2 | -0.8±0.2 | -1.4 |
| ASN60 N | SER56 O | 0.71 ± 0.12 | -5.7 ± 0.3 | -6.1±0.2 | 0.4 |
| LEU61 N | PHE58 O | 0.88 ± 0.05 | 3.2 ± 0.2 | 1.2±0.2 | 2.1 |
| GLN66 N | PRO62 O | 0.99 ± 0.01 | -4.9 ± 0.2 | -3.9±0.2 | -0.9 |
| GLN67 N | LEU63 O | 1.00 ± 0.00 | 0.6 ± 0.2 | 1.1±0.3 | -0.5 |
| HIS68 N | TRP64 O | 0.99 ± 0.01 | 2.6 ± 0.2 | 3.8±0.2 | -1.2 |
| LEU69 N | LYS65 O | 0.99 ± 0.01 | 0.4 ± 0.2 | 1.5±0.2 | -1.1 |
| LYS70 N | GLN66 O | 0.99 ± 0.01 | 0.7 ± 0.2 | 1.7±0.2 | -1.0 |
| LYS71 N | GLN67 O | 0.99 ± 0.01 | 0.4 ± 0.3 | 1.8±0.2 | -1.4 |
| GLU72 N | HIS68 O | 0.99 ± 0.01 | -2.0 ± 0.3 | -0.9±0.2 | -1.1 |
| LYS73 N | LEU69 O | 0.95 ± 0.03 | 1.6 ± 0.2 | 1.3±0.4 | 0.3 |
| GLY74 N | LYS71 O | 0.85 ± 0.08 | 8.3 ± 0.2 | 8.5±0.2 | -0.3 |
| LEU75 N | LYS70 O | 0.93 ± 0.05 | -1.7 ± 0.3 | -0.9±0.3 | -0.8 |
|  |  | **Mean** | **-10.16** | **-9.37** | **-0.8** |

**Table S1 –** The mean and standard deviation of the interaction energy for individual hydrogen bonds in the native and extended states for the CHARMM22/TIP3P/NaCl treatment, plus the energy difference. In each case, the donor and acceptor heavy atoms are reported, using residue names, residue numbers, and CHARMM atom names.

| **Atom 1** | **Atom 2** | **Persistence** | **Native**  **E_NP_ (kcal/mol)** | **Extended E_NP_ (kcal/mol)** | **ΔE_NP_ (kcal/mol)** |
| --- | --- | --- | --- | --- | --- |
| PHE47 CZ | PHE58 CE1/PHE58 CE2 | 0.51 ± 0.16 | 1.8 ± 0.1 | 2.0 ± 0.1 | -0.2 |
| LEU42 CB | ASP46 CB | 0.53 ± 0.20 | -24.8 ± 0.1 | -24.5 ± 0.1 | -0.3 |
| PHE51 CE1/PHE51 CE2 | LYS70 CG | 0.54 ± 0.08 | 6.9 ± 0.1 | 7.2 ± 0.1 | -0.4 |
| TRP64 CE3 | LYS65 CG | 0.55 ± 0.19 | 6.2 ± 0.1 | 6.8 ± 0.1 | -0.6 |
| LYS71 CG | PHE76 CD1/PHE76 CD2 | 0.56 ± 0.21 | 4.2 ± 0.1 | 4.5 ± 0.1 | -0.3 |
| VAL50 CB | PHE51 CE1/PHE51 CE2 | 0.57 ± 0.15 | 4.2 ± 0.1 | 4.5 ± 0.1 | -0.3 |
| MET53 CG | PHE58 CB | 0.58 ± 0.09 | -3.3 ± 0.1 | -2.1 ± 0.1 | -1.2 |
| PHE47 CE1/PHE47 CE2 | PHE51 CB | 0.58 ± 0.17 | -1.0 ± 0.1 | -0.4 ± 0.1 | -0.6 |
| PHE47 CZ | PHE58 CG | 0.59 ± 0.17 | 1.6 ± 0.1 | 1.7 ± 0.1 | -0.2 |
| TRP64 CH2 | LYS65 CG | 0.59 ± 0.16 | 7.2 ± 0.1 | 7.9 ± 0.1 | -0.7 |
| LYS70 CB | PHE76 CD1/PHE76 CD2 | 0.59 ± 0.12 | -1.4 ± 0.1 | -1.0 ± 0.1 | -0.4 |
| TRP64 CZ3 | LYS65 CG | 0.60 ± 0.13 | 6.0 ± 0.1 | 6.7 ± 0.1 | -0.7 |
| TRP64 CZ2 | LYS65 CG | 0.60 ± 0.21 | 1.0 ± 0.1 | 1.6 ± 0.1 | -0.6 |
| TRP64 CD2 | LYS65 CG | 0.60 ± 0.22 | 4.7 ± 0.1 | 5.4 ± 0.1 | -0.7 |
| LYS73 CB | LEU75 CG | 0.61 ± 0.11 | 3.4 ± 0.1 | 3.7 ± 0.1 | -0.2 |
| PHE58 CD1/PHE58 CD2 | LEU61 CD1/LEU61 CD2 | 0.61 ± 0.10 | -9.6 ± 0.1 | -8.7 ± 0.1 | -1.0 |
| VAL50 CG1/VAL50 CG2 | PHE51 CZ | 0.62 ± 0.12 | -2.9 ± 0.1 | -2.7 ± 0.1 | -0.2 |
| PHE58 CD1/PHE58 CD2 | LEU69 CD1/LEU69 CD2 | 0.62 ± 0.08 | -9.4 ± 0.1 | -8.7 ± 0.1 | -0.7 |
| LEU61 CD1/LEU61 CD2 | GLN66 CG | 0.62 ± 0.16 | -20.2 ± 0.1 | -19.0 ± 0.1 | -1.2 |
| LEU61 CD1/LEU61 CD2 | LYS65 CD | 0.63 ± 0.15 | -11.8 ± 0.1 | -11.4 ± 0.1 | -0.3 |
| ASP44 CB | THR54 CG2 | 0.63 ± 0.19 | -32.7 ± 0.1 | -31.8 ± 0.1 | -0.9 |
| MET53 SD | PHE58 CB | 0.64 ± 0.05 | -7.1 ± 0.1 | -5.7 ± 0.1 | -1.4 |
| PHE51 CG | PHE58 CE1/PHE58 CE2 | 0.64 ± 0.13 | 2.4 ± 0.1 | 2.7 ± 0.1 | -0.3 |
| PHE51 CD1/PHE51 CD2 | PHE58 CZ | 0.64 ± 0.14 | -0.6 ± 0.1 | -0.6 ± 0.1 | 0.0 |
| LEU61 CG | LYS65 CB | 0.65 ± 0.36 | 3.0 ± 0.1 | 3.7 ± 0.1 | -0.7 |
| PHE51 CD1/PHE51 CD2 | LEU69 CD1/LEU69 CD2 | 0.66 ± 0.15 | -9.0 ± 0.1 | -8.7 ± 0.1 | -0.3 |
| PHE47 CZ | ARG55 CG | 0.66 ± 0.07 | -4.4 ± 0.1 | -3.9 ± 0.1 | -0.5 |
| MET53 CG | ALA57 CB | 0.67 ± 0.08 | -0.8 ± 0.1 | 0.3 ± 0.1 | -1.1 |
| MET53 SD | ALA57 CB | 0.68 ± 0.06 | -4.6 ± 0.1 | -3.3 ± 0.1 | -1.3 |
| GLN67 CG | PHE76 CE1/PHE76 CE2 | 0.68 ± 0.21 | -10.1 ± 0.1 | -9.8 ± 0.1 | -0.3 |
| MET53 CE | LEU69 CD1/LEU69 CD2 | 0.71 ± 0.07 | -11.2 ± 0.1 | -10.8 ± 0.1 | -0.5 |
| MET53 SD | PHE58 CD1/PHE58 CD2 | 0.71 ± 0.06 | -5.8 ± 0.1 | -4.9 ± 0.1 | -0.9 |
| VAL50 CG1/VAL50 CG2 | PHE51 CG | 0.72 ± 0.10 | -2.6 ± 0.1 | -2.1 ± 0.1 | -0.5 |
| PHE58 CZ | GLN66 CB | 0.74 ± 0.16 | 8.9 ± 0.1 | 8.9 ± 0.1 | 0.0 |
| LEU61 CB | GLN66 CG | 0.75 ± 0.16 | -17.2 ± 0.1 | -16.1 ± 0.1 | -1.0 |
| LYS73 CB | LEU75 CD1/LEU75 CD2 | 0.78 ± 0.11 | -7.8 ± 0.1 | -7.4 ± 0.1 | -0.4 |
| LEU61 CD1/LEU61 CD2 | LEU69 CD1/LEU69 CD2 | 0.79 ± 0.09 | -16.2 ± 0.1 | -15.1 ± 0.1 | -1.1 |
| PHE47 CE1/PHE47 CE2 | PHE51 CG | 0.80 ± 0.23 | 2.1 ± 0.1 | 2.7 ± 0.1 | -0.5 |
| PHE51 CD1/PHE51 CD2 | PHE58 CD1/PHE58 CD2 | 0.80 ± 0.16 | -2.5 ± 0.1 | -2.3 ± 0.1 | -0.3 |
| PHE47 CG | PHE51 CD1/PHE51 CD2 | 0.83 ± 0.05 | -0.2 ± 0.1 | 0.2 ± 0.1 | -0.4 |
| PHE51 CE1/PHE51 CE2 | PHE58 CZ | 0.83 ± 0.15 | 2.1 ± 0.1 | 2.1 ± 0.1 | 0.0 |
| VAL50 CG1/VAL50 CG2 | LEU75 CD1/LEU75 CD2 | 0.85 ± 0.19 | -11.2 ± 0.1 | -10.7 ± 0.1 | -0.5 |
| PHE47 CB | ARG55 CG | 0.86 ± 0.14 | -7.4 ± 0.1 | -6.5 ± 0.1 | -0.9 |
| VAL50 CB | PHE51 CD1/PHE51 CD2 | 0.87 ± 0.19 | 1.4 ± 0.1 | 1.9 ± 0.1 | -0.5 |
| LEU42 CD1/LEU42 CD2 | ASP46 CB | 0.88 ± 0.15 | -29.8 ± 0.1 | -29.4 ± 0.1 | -0.4 |
| PHE51 CZ | LEU75 CD1/LEU75 CD2 | 0.88 ± 0.06 | -7.2 ± 0.1 | -6.9 ± 0.1 | -0.3 |
| PHE58 CZ | GLN66 CG | 0.89 ± 0.05 | -11.5 ± 0.1 | -10.9 ± 0.1 | -0.6 |
| PHE58 CD1/PHE58 CD2 | GLN66 CG | 0.90 ± 0.08 | -13.3 ± 0.1 | -12.6 ± 0.1 | -0.7 |
| MET53 CB | ALA57 CB | 0.90 ± 0.07 | 1.2 ± 0.1 | 1.8 ± 0.1 | -0.6 |
| PHE47 CE1/PHE47 CE2 | PHE58 CG | 0.91 ± 0.05 | 2.5 ± 0.1 | 2.8 ± 0.1 | -0.3 |
| PHE47 CE1/PHE47 CE2 | PHE58 CB | 0.91 ± 0.05 | -1.4 ± 0.1 | -0.4 ± 0.1 | -1.0 |
| PHE58 CE1/PHE58 CE2 | LEU69 CD1/LEU69 CD2 | 0.92 ± 0.03 | -6.4 ± 0.1 | -6.0 ± 0.1 | -0.5 |
| PHE47 CZ | PHE51 CD1/PHE51 CD2 | 0.93 ± 0.17 | -1.0 ± 0.1 | -0.6 ± 0.1 | -0.4 |
| PHE47 CZ | PHE51 CE1/PHE51 CE2 | 0.93 ± 0.10 | 1.8 ± 0.1 | 2.1 ± 0.1 | -0.3 |
| PHE47 CG | ARG55 CG | 0.96 ± 0.10 | -3.7 ± 0.1 | -3.2 ± 0.1 | -0.5 |
| PHE58 CE1/PHE58 CE2 | GLN66 CB | 0.97 ± 0.23 | 9.9 ± 0.1 | 9.9 ± 0.1 | 0.0 |
| PHE47 CD1/PHE47 CD2 | PHE51 CE1/PHE51 CE2 | 0.98 ± 0.07 | 0.1 ± 0.1 | 0.4 ± 0.1 | -0.3 |
| PHE47 CZ | PHE58 CD1/PHE58 CD2 | 0.98 ± 0.28 | -0.9 ± 0.1 | -0.6 ± 0.1 | -0.3 |
| MET53 CE | LEU61 CD1/LEU61 CD2 | 1.10 ± 0.14 | -11.4 ± 0.1 | -10.7 ± 0.1 | -0.7 |
| LEU61 CD1/LEU61 CD2 | LYS65 CB | 1.11 ± 0.49 | -8.4 ± 0.1 | -7.5 ± 0.1 | -0.9 |
| PHE51 CE1/PHE51 CE2 | LEU75 CD1/LEU75 CD2 | 1.13 ± 0.10 | -6.1 ± 0.1 | -5.9 ± 0.1 | -0.3 |
| MET53 SD | LEU61 CD1/LEU61 CD2 | 1.14 ± 0.12 | -12.6 ± 0.1 | -11.2 ± 0.1 | -1.4 |
| PHE47 CE1/PHE47 CE2 | PHE58 CD1/PHE58 CD2 | 1.22 ± 0.12 | 0.0 ± 0.1 | 0.4 ± 0.1 | -0.4 |
| PHE51 CE1/PHE51 CE2 | PHE58 CE1/PHE58 CE2 | 1.25 ± 0.22 | 3.1 ± 0.1 | 3.1 ± 0.1 | 0.0 |
| VAL50 CG1/VAL50 CG2 | PHE51 CE1/PHE51 CE2 | 1.28 ± 0.18 | -2.0 ± 0.1 | -1.7 ± 0.1 | -0.3 |
| VAL50 CG1/VAL50 CG2 | PHE51 CD1/PHE51 CD2 | 1.31 ± 0.16 | -4.7 ± 0.1 | -4.4 ± 0.1 | -0.4 |
| PHE51 CD1/PHE51 CD2 | PHE58 CE1/PHE58 CE2 | 1.34 ± 0.20 | 0.4 ± 0.1 | 0.4 ± 0.1 | 0.0 |
| PHE58 CE1/PHE58 CE2 | GLN66 CG | 1.48 ± 0.12 | -10.5 ± 0.1 | -9.9 ± 0.1 | -0.6 |
| PHE47 CE1/PHE47 CE2 | PHE51 CE1/PHE51 CE2 | 1.51 ± 0.08 | 2.9 ± 0.1 | 3.1 ± 0.1 | -0.2 |
| PHE47 CE1/PHE47 CE2 | ARG55 CG | 1.57 ± 0.12 | -3.4 ± 0.1 | -2.9 ± 0.1 | -0.5 |
| PHE47 CD1/PHE47 CD2 | PHE51 CD1/PHE51 CD2 | 1.60 ± 0.13 | -2.8 ± 0.1 | -2.3 ± 0.1 | -0.5 |
| PHE47 CE1/PHE47 CE2 | PHE51 CD1/PHE51 CD2 | 1.76 ± 0.22 | 0.1 ± 0.1 | 0.4 ± 0.1 | -0.3 |
| PHE47 CD1/PHE47 CD2 | ARG55 CG | 1.86 ± 0.15 | -6.1 ± 0.1 | -5.6 ± 0.1 | -0.5 |
|  |  |  | **-4.0** | **-3.5** | **-0.5** |

**Table S2 –** The mean and standard deviation of the interaction energy for non-polar contacts in the native and extended states for the CHARMM22/TIP3P/NaCl treatment, plus the energy difference. In each case, the two non-polar heavy atoms are reported, using residue names, residue numbers, and CHARMM atom names.
